# Perfluorooctanoic Acid Exposure Assessment on Common Carp Liver through Image and Ultrastructural Investigation

**DOI:** 10.3390/ijerph16244923

**Published:** 2019-12-05

**Authors:** Maurizio Manera, Bahram Sayyaf Dezfuli, Giuseppe Castaldelli, Joseph A. DePasquale, Elisa Anna Fano, Camillo Martino, Luisa Giari

**Affiliations:** 1 Faculty of Biosciences, Food and Environmental Technologies, University of Teramo, St. R. Balzarini 1, 64100 Teramo, Italy; 2 Department of Life Sciences and Biotechnology, University of Ferrara, St. Borsari 46, 44121 Ferrara, Italy; dzb@unife.it (B.S.D.); giuseppe.castaldelli@unife.it (G.C.); anna.fano@unife.it (E.A.F.); luisa.giari@unife.it (L.G.); 3 Morphogenyx Inc., PO Box 717, East Northport, NY 11731, USA; jadepasquale@morphogenyx.com; 4 Department of Veterinary, University of Perugia, St. San Costanzo 4, 06126 Perugia, Italy; camillo.martino92@gmail.com

**Keywords:** grayscale differential box counting, fractal analysis, complexity, cloudy swelling, endoplasmic reticulum stress, hormesis

## Abstract

Perfluorooctanoic acid (PFOA) poses particular concern as an emerging pollutant in both surface and ground waters. Fish, as a natural inhabitant of these waters and being highly representative of vertebrates, represents an ideal animal model to assess the toxic effects of PFOA. Hereby, liver microscopic texture was comparatively evaluated in individuals of common carp subchronically exposed to PFOA using grayscale differential box counting, a fractal analysis method. Furthermore, liver cytoplasmic glycogen areas and ultrastructure were also evaluated and compared to the image analysis findings. Redundancy Analysis was performed to assess, in summary, how much the variation of fractal dimension and lacunarity was explained by the concentration of PFOA in liver, the mass of liver and the number of proliferating cell nuclear antigen (PCNA)-immunoreactive nuclei. Treatment group ordination was better determined by fractal dimension than lacunarity. Interestingly, a significant complexity increase was associated with the modification of liver microscopic texture due to PFOA exposure. This complexity increase was related to “cloudy swelling”, possibly representing a primarily adaptive strategy against PFOA challenge, rather than a slight, reversible form of degeneration as traditionally proposed. The occurrence of endoplasmic reticulum stress, unfolded protein reaction and hormetic response was proposed and discussed.

## 1. Introduction

Perfluorinated alkylated substances (PFASs) are widely used industrial organic chemicals favored for their high stability and resistance to degradation. However, it is these very same features that have made PFASs a rising global concern, as these compounds continue to accumulate in the environment [1,2,3,4,5].

Among PFASs, perfluorooctanoic acid (PFOA) poses particular concern as an emerging pollutant because of its water and oil repellence, heat resistance and chemical stability—properties favorable to its use in a wide range of technical, industrial uses, ranging from fluoropolymers, fabrics, cosmetics, detergents, fire-fighting foams, to food packaging [2,4,5]. Wide distribution, environmental persistence, bioconcentration/bioaccumulation in organisms, biomagnification across food webs, possible toxicity, carcinogenicity and endocrine disruption in animals are the main concerns for PFOA [1,4,6,7]. Indeed, PFOA can be found in surface and ground waters, in animals and human beings, even in remote, unurbanized areas [8,9,10]. The biotransformation of PFASs is insignificant or absent [11,12], indicating other mechanisms must underlie the removal from the organism of such substances. In particular, surface-active properties can influence the enterohepatic recirculation of PFOA and other PFASs, accounting for their slow depuration rate [11,13]. Toxicological and ecotoxicological studies of PFASs mainly rely on the liver as a sensitive target of pollutants because of this organ’s crucial role in animal metabolism and ultimately in the entire health of the organism [1,14,15,16]. Fish are the most representative vertebrates and are thus widely used as models in biomedical, toxicological and ecotoxicological research and in biomonitoring programs [17,18,19,20,21,22]. Fish models are particularly relevant to the study of PFASs which are continuously introduced into waters [23,24,25,26,27]. Both gene expression and enzyme activity are altered in fish liver as a consequence of PFOA exposure, according to species and exposure regimen [7,28,29,30,31,32]. 

In a previous study [15], individual samples were analyzed with a combination of texture analysis and multivariate exploratory data analysis, resulting in a reliable method to investigate the liver microscopic modification related to PFOA exposure. This combined analysis yielded higher confidence in the results as well as a more economical approach compared to PFOA analytical determination. In particular, the selected texture features identified with confidence liver microscopic modification according to treatment, correlating well with the analytical, hepatic PFOA concentrations, so that the overall approach may represent an innovative, robust, sufficiently sensible and sensitive biomarker of exposure and effect [15]. Furthermore, as an alternative means to assess image texture, the grayscale differential box counting method was adopted for fractal analysis. Results from this approach were compared to previous outcomes and an increase in the complexity associated with a decrease in the disorder of the image grayscale pattern was observed, though the biological significance remained unanswered [33].

Traditional histopathology has been successfully used in toxicological and ecotoxicological studies in fish [22,34,35,36]. However, because it relies on trained human operators, it may be affected by errors. Furthermore, it is not a quantitative discipline and this fact partially impair the adoption of full statistical analysis on its descriptive and primarily qualitative results [37,38,39]. Consequently, researchers are looking for possible objective, replicable methods to reduce and ideally avoid the occurrence of human-dependent errors [40,41,42,43,44] and the application of image analysis tools to histopathology aims to achieve these goals [15,18,42,45,46,47].

The present survey used image analysis to assess carp liver pathogenesis after exposure to PFOA. Images of liver tissue were evaluated with respect to the relationship between liver pathology and immunohistochemical data (Proliferating Cell Nuclear Antigen (PCNA)), chemical data (PFOA liver concentration), liver cytoplasmic glycogen areas, and ultrastructure. The results suggest a possible involvement of endoplasmic reticulum stress and, consequently, of unfolded protein response as a biphasic/hormetic reaction which is initially compensatory, adaptive, and finally truly degenerative as previously supposed [48].

To date, no previous published research focused on the ultrastructural liver pathology as a consequence of PFOA exposure in fish.

## 2. Materials and Methods

The experimental design, the immunohistochemical and ultrastructural methods are only briefly summarized here, because they have already been reported in detail elsewhere [14].

### 2.1. Fish Selection and Subchronic Exposure

Thirty exemplars of common carp (*Cyprinus carpio* Linnaeus, 1758) were randomly allocated in 3 groups (10 fish in a control tank; 10 fish in a low dosage exposure tank [200 ng L^−1^ PFOA]; 10 fish in a high dosage exposure tank [2 mg L^−1^ PFOA]) and treated according to Organization for Economic Co-operation and Development guidelines [49]. A flow-through system was adopted to maintain the previously reported PFOA experimental concentrations for the duration of the test (56 days). The tested concentrations were chosen based on previous reports of PFOA occurrence in surface waters [50,51] and on previous toxicological reports [29,52]. At the end of the trial fish were sacrificed (pithed) after anesthesia with buffered solution of tricaine methanesulfonate and the following parameters were obtained from each exemplars: total length, body mass and liver mass. PFOA concentration in liver was also evaluated for each fish as previously reported [14].

### 2.2. Tissue Processing for Light, Transmission Electron Microscopy and Histological Observation

Samples were obtained from the liver of each fish, paying attention to include both the periphery and the core of the organ and to rule out gross pathology. After fixation in 10% neutral buffered formalin, the samples were dehydrated, clarified, paraffin embedded, sectioned at 5 µm and stained with hematoxylin and eosin (H&E).

Indirect immunohistochemistry was adopted on tissue sections to test proliferative cell nuclear antigen (PCNA) immunoreactivity according to Dezfuli and colleagues (2012) [53].

A bright field microscope equipped with a digital color camera was adopted to observe the previous and the following tissue sections. Tissue sections were observed at 200× total magnification and images from homogeneous parenchimatous tissue area were recorded in uncompressed TIFF file format for texture analysis. The number of PCNA-immunoreactive nuclei was obtained by screening 2 randomly selected field per fish at 400× total magnification and counting 1000 hepatocyte nuclei. 

Samples of liver were also fixed in buffered 2% glutaraldehyde and routinely processed for transmission electron microscopy to obtained ultrathin sections (90 nm). They were stained with toluidine blue and were examined at light microscopy at 1000× total magnification.

Glycogen percentage cytoplasmic area was assessed, according to treatment groups as follows. The TIFF color images were binarized using the “Threshold Color” plugin of ImageJ (v1.52r; Rasband W., National Institute of Health, Bethesda, MD, USA). “Triangle” was adopted as thresholding method and background was set to dark. The resulting segmented glycogen cytoplasmic areas were reported as percentage with respect to total area in pixel.

For transmission electron microscopy, ultrathin sections were stained with uranyl acetate and lead citrate, then observed and photographed with Zeiss EM 910 (Zeiss, Oberkochen, Germany) electron microscope operating at 120 kV.

### 2.3. Fractal Analysis

Fractal dimension and lacunarity were computed on grayscale-converted images using the FracLac Image J plugin [54] with the grayscale differential box counting method, at the default setting, to preserve grayscale texture information as previously reported [33,48].

### 2.4. Statistical Analysis

All numerical data (fractal dimension, lacunarity and glycogen percentage cytoplasmic area in pixel) were assessed for normality and homogeneity of variance. Thereafter, Generalized Linear Model (GLM) was used to test for significant differences among treatment groups, adopting SPSS^®^ 14.0.2 (SPSS Inc., Chicago, IL, USA) as statistical software.

Redundancy Analysis (RDA) was performed using Canoco 5.12 [55] to assess, in summary, how much the variation of fractal dimension and lacunarity was explained by the concentration of PFOA in liver, by the mass of liver and by the number of proliferating cell nuclear antigen (PCNA)-immunoreactive nuclei. Specifically, fractal dimension and lacunarity were introduced as main matrix data, while the following biometric/experimental variables were introduced as secondary matrix data: liver mass, the number of PCNA-immunoreactive nuclei, and PFOA liver concentration. Total length was used as covariate.

## 3. Results

**Figure 1:**
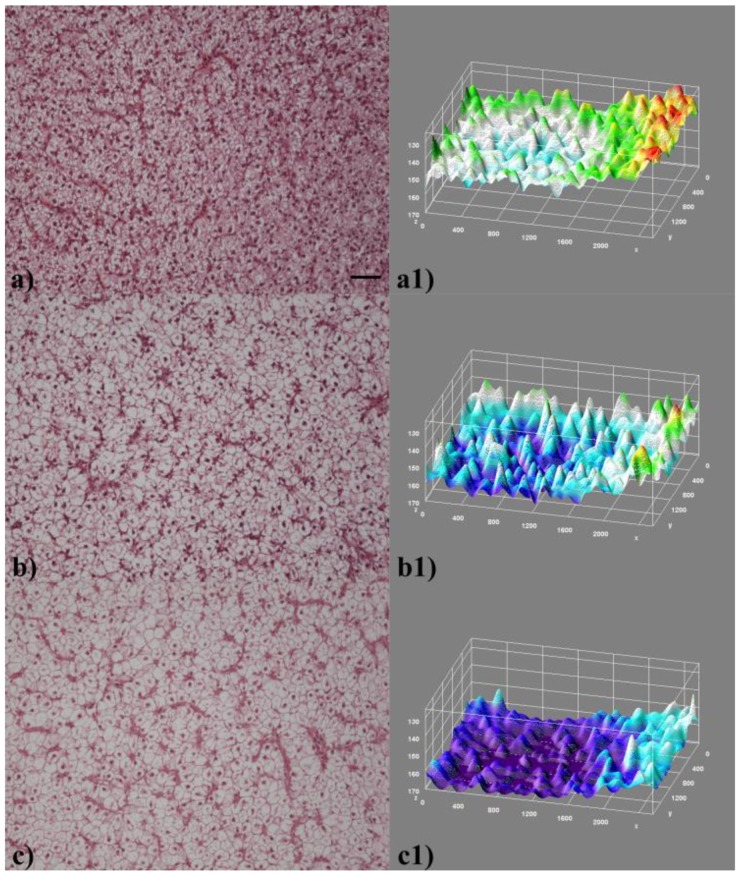
Liver histological sections according to treatment: (**a**) Control (Ctr), (**b**) Low dosage perfluorooctanoic acid (PFOA), and (**c**) High dosage PFOA. Hematoxylin and eosin. Bar = 50 µm. Corresponding pseudo-3D landscape images are shown in (**a1**–**c1**), where grayscale values (z axis) are inverted—namely, 0 (black) is at the top of the scale, while 255 (white) is at the bottom of the scale—and are reported in color code (therefore, violet values correspond to higher grayscale values than do red values).

The microscopic appearance of representative sections of liver tissue, according to each treatment group, is shown in Figure 1. In the liver of PFOA-treated fish (Figure 1b,c), hepatocytes appeared enlarged and clearer while hepatic sinusoid was less visible compared to untreated, control fish (Figure 1a), as a result of the compression elicited by the enlarged, degenerated hepatocytes.

The grayscale differential box counting results are summarized in Table 1.

Both fractal dimension and lacunarity differed significantly among treatment groups, with the exception of data from low dosage-treated fish, which did not differ from those of untreated fish.

The results of biometric (liver mass), immunohistochemical (number of PCNA-immunoreactive nuclei) and analytical data (liver PFOA concentration) are summarized in Table 2.

**Figure 2:**
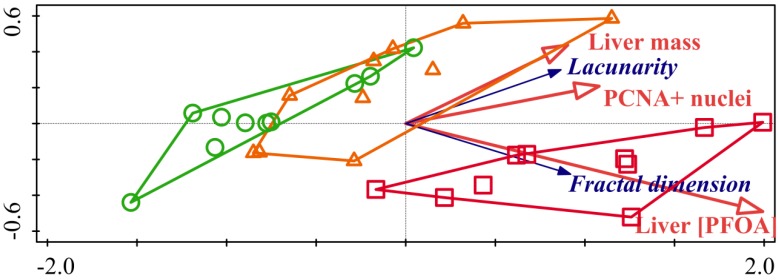
Redundancy Analysis (RDA) ordination tri-plot of grayscale differential box counting features and biometric (liver mass, number of PCNA-immunoreactive nuclei) and analytical (liver PFOA concentrations) data. Fractal dimension and lacunarity are reported as blue vectors, whereas liver mass, number of PCNA-immunoreactive nuclei and liver PFOA concentration as red vectors. The partial superimposing of the Ctr and Low dosage PFOA convex hulls is visible (●, Ctr; ▲ Low PFOA; ■ High PFOA).

The RDA ordination tri-plot of fractal analysis data (fractal dimension and lacunarity), biometric (liver mass and total length as covariate), immunohistochemical (number of PCNA-immunoreactive nuclei) and analytical data (liver PFOA concentration) is shown in Figure 2.

**Figure 3:**
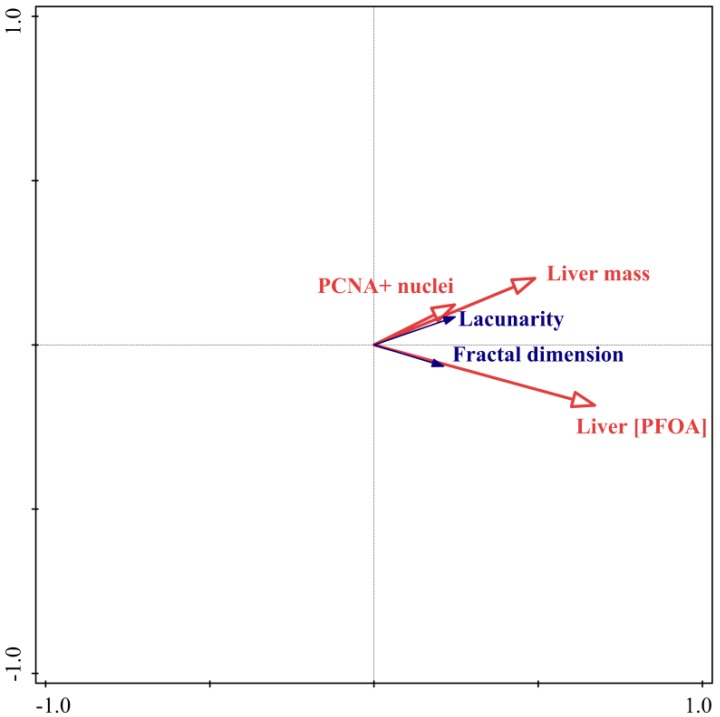
RDA *t*-value bi-plot. Fractal dimension and lacunarity are reported as blue vectors, whereas liver mass, number of PCNA-immunoreactive nuclei and liver PFOA concentration as red vectors.

**Figure 4:**
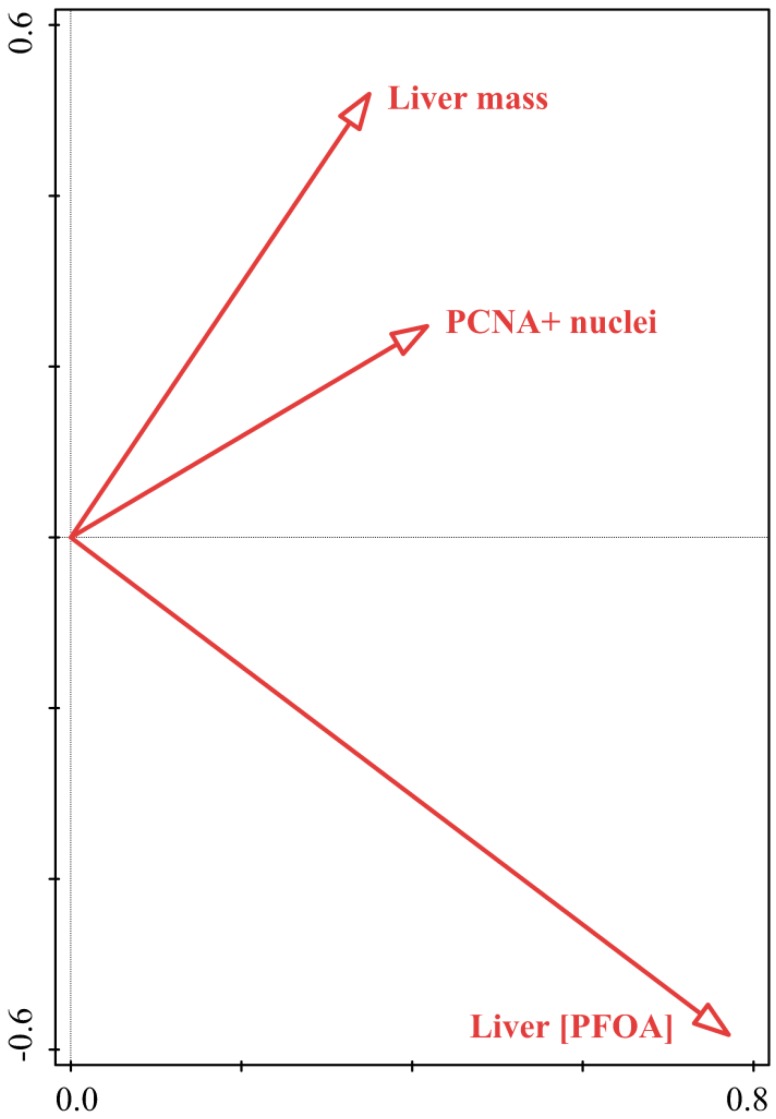
RDA plot of liver mass, number of PCNA-immunoreactive nuclei, and liver PFOA concentration.

The cumulative percentage variance of response data and of fitted response data for axis 1 and 2 was, respectively, 66.0 and 66.8 and 98.9 and 100.0. The test of significance of all canonical axes (axes 1 and 2) showed a *p* value < 0.01. The percentage contribution of the selected second matrix variables was: PFOA liver concentration, 61.6 (*p* < 0.01); liver mass, 28.3 (*p* < 0.01); PCNA-immunorective nuclei, 7.5 (*p* = 0.06). Only cases from the high dosage group did not show any convex hull superimposing, being regrouped only in the 4th Cartesian quadrant (with the exception of one case). The contrasting cases from untreated and low dosage-treated fish were regrouped in all the first three Cartesian quadrants, with a partial superimposing of the relative convex hulls (Figure 2). This ordination result is confirmed by Anova, reporting no significant differences between fractal dimension and lacunarity values from untreated fish compared to low dosage-treated fish (Table 1). Because the score scaling of the previous reported ordination tri-plot is of compromise type (with standardized grayscale differential box counting data scores), the *t*-value bi-plot (Figure 3) and the second matrix variables (biometric [liver mass and total length as covariate], immunohistochemical [PCNA-immunoreactive nuclei] and analytical data [liver PFOA concentration]) plot (Figure 4) are also shown.

With regard to the *t*-value bi-plot (Figure 3), it estimates the *t*-values of the regression coefficients, from the multiple regression with a particular grayscale differential box counting feature, as the response variable, and the second matrix variables (biometric [liver mass and total length as covariate], immunohistochemical [PCNA-immunoreactive nuclei] and analytical data [liver PFOA concentration]) as predictors. The arrowheads, of the latter variables can be projected on the ideal line overlapping the arrow of a specific grayscale differential box counting feature, so that, if such projection lies on this ideal line further from the arrowhead of that grayscale differential box counting feature, the corresponding *t*-value of regression coefficient is deduced to have a value larger than 2. This held true for both the regression coefficient of liver PFOA concentration and of liver mass with respect to fractal dimension and lacunarity and for the regression coefficient of the number of PCNA-immunoreactive nuclei only with respect to lacunarity, though the *t*-value of the regression with fractal dimension approaches that value (Figure 3).

Considering Figure 4, each arrow is direct toward the sharpest increase in second matrix variable values. The correlation between second matrix variables can be estimated from the angle formed by each couple of arrows. In particular, correlations of an individual second matrix variable with the other variables can be estimated by projecting their arrowheads on the ideal line overlaying that variable’s arrow. Moreover, the coordinates of each arrow tip from the second matrix variables are correlations of the corresponding variable with the ordination axes. Therefore, liver PFOA concentration correlated with PCNA-immunoreactive nuclei and did not correlate with liver mass. On the contrary, PCNA-immunoreactive nuclei correlated with liver mass (Figure 4).

**Figure 5:**
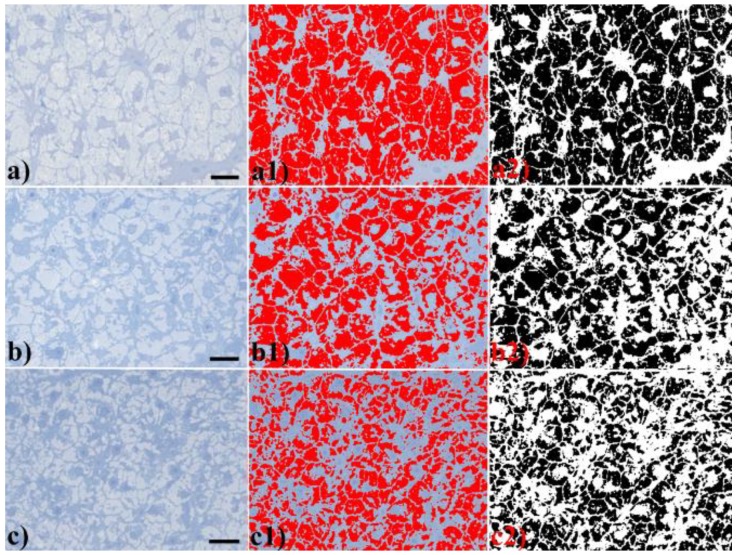
Liver structure according to treatment group: (**a**) Ctr, (**b**) Low dosage PFOA, and (**c**) High dosage PFOA. Hepatocytes from the control group showed a prominent cytoplasmic clearer area, corresponding to glycogen area. A portion of darker protoplasm could be appreciated as a thin rim around the nuclei. In PFOA-treated fish, the cytoplasmic glycogen areas were percentually reduced with respect to the darker cytoplasm portions that increased according to PFOA dosage. Moreover, with PFOA treatment, the darker portion of the protoplasm showed vacuolizations. Ultrathin sections. Toluidine blue. Bar = 10 µm. The color thresholding procedure was depicted in (**a1**–**c1**), showing the red superimposed glycogen areas. Finally corresponding segmented areas are shown in (**a2**–**c2**).

Light microscopy evaluation of liver ultrathin sections established the cytoplasm modifications in hepatocytes according to treatment group (Figure 5). In particular, hepatocytes from the control group showed a prominent cytoplasmic clearer area (Figure 5a) corresponding to the cytoplasmic glycogen areas seen with the Periodic Acid–Schiff (PAS) reaction in previous research [15]. A portion of darker cytoplasm could be seen as a thin rim around the nuclei (Figure 5a). In contrast, in PFOA-treated fish, the cytoplasmic clearer areas were percentually reduced with respect to total tissue areas and to the darker cytoplasm portions that increased according to PFOA dosage (compare Figure 5a with Figure 5b,c). Moreover, the darker portion of the cytoplasm showed vacuolizations according to treatment. The adoption of image analysis techniques permitted an unbiased analysis of this cytoplasmic area, which was disproportionate with respect to treatment, as shown in Figure 5a1–c1,a2–c2.

**Figure 6:**
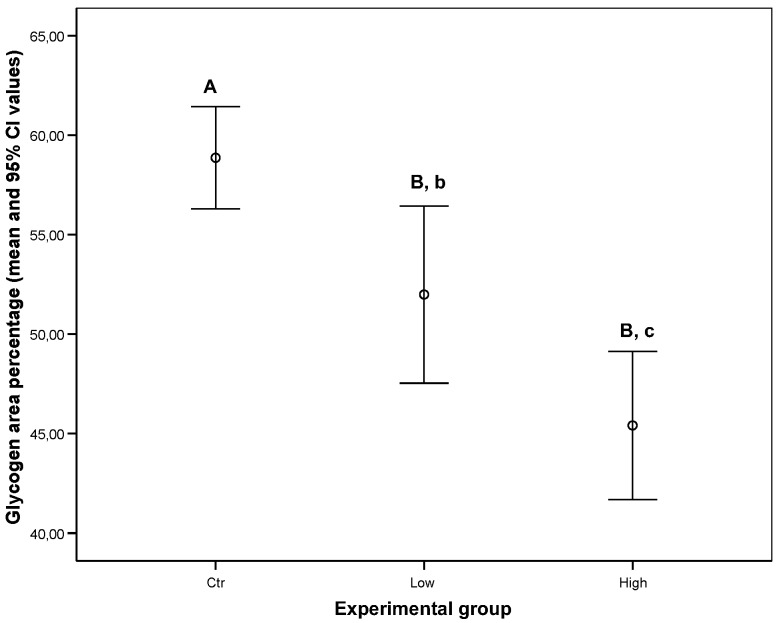
Mean and 95% confidence intervals of glycogen area percentage (with respect to total area in pixel) according to treatment. Means with different letters differ significantly. In particular, uppercase letters highlight *p* < 0.01 (Anova) and are significant differences; lowercase letters highlight *p* < 0.05 (Anova) and are significant differences.

In particular, all groups differed significantly among each other (ctr vs. low dosage PFOA, Anova *p* < 0.01; ctr vs. high dosage PFOA, Anova *p* < 0.01; low dosage PFOA vs. high dosage PFOA, Anova *p* < 0.05) (Figure 6).

**Figure 7:**
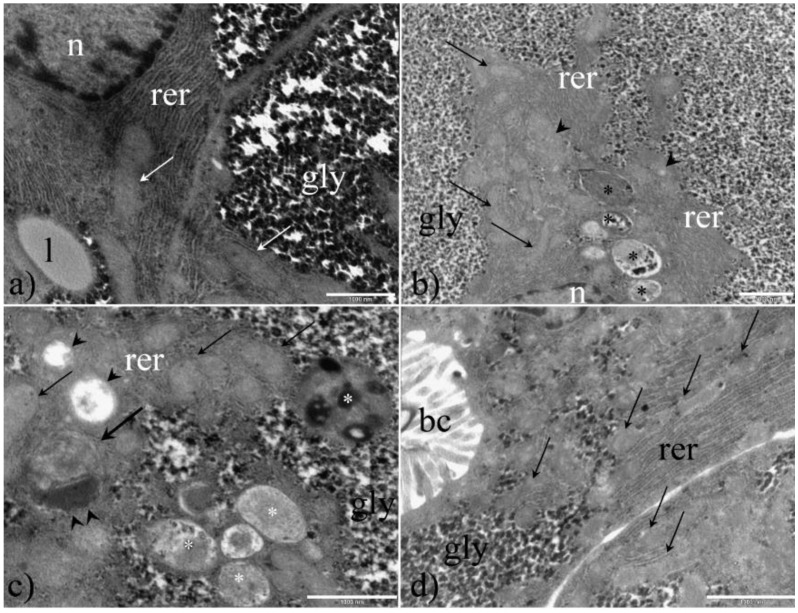
Liver ultrastructure according to treatment group: (**a**) Ctr, (**b**,**c**) Low dosage PFOA, and (**d**) High dosage PFOA. Ctr fish (**a**) showed a clear compartmentation of the cytoplasm with a wide area replete with densely packed glycogen particles (gly) and a smaller perinuclear cytoplasmic area containing principally rough endoplasmic reticulum (rer), mitochondria (white thin arrows) and lipid droplets (l). PFOA treatment affected hepatocyte ultrastructure according to the dosage. In particular, the perinuclear cytoplasmic area appeared enlarged compared to the glycogen area (gly); the rough endoplasmic reticulum (rer) was enlarged and showed dilated cisternae, replete with flocculent content (black thin arrows in **d**). Moreover, mitochondria showed alteration ranging from ballooned (arrowheads in **b**) to disintegrated cristae and matrix vacuolization (arrowheads in **c**). Autophagosomes were also appreciable (black and white asterisks in **b** and **c**, respectively), together with myelin figure formation (thick arrow and double arrowheads in **c**). N = nucleus; bc = bile canaliculus. Bar = 1000 nm.

Examination of hepatocyte ultrastructure in unexposed fish showed a clear compartmentation of the cytoplasm with a wide area replete with densely packed glycogen particles and a smaller perinuclear cytoplasmic area containing principally rough endoplasmic reticulum, mitochondria and lipid droplets (Figure 7a). PFOA treatment affected hepatocyte ultrastructure according to the dosage. In contrast to control hepatocytes the perinuclear cytoplasm of PFOA-exposed hepatocytes appeared enlarged compared to the glycogen area, rough endoplasmic reticulum was also enlarged and showed dilated cisternae, replete with flocculent content (Figure 7b–d). Moreover, mitochondria showed structural changes ranging from ballooned to disintegrated cristae and matrix vacuolization. Autophagosomes were also appreciable, according to the recommendations of Eskelinen (2008) [56], together with myelin figure formation (Figure 7b,c).

## 4. Discussion

The pathological changes observed in PFOA-treated liver, using both light and electron microscopy, are attributable to cloudy swelling. From a historical perspective, cloudy swelling (trübe Schwellung according to Virchow) has been considered as a slight, reversible form of cell degeneration [57,58]. In particular, it was observed as an increase in cellular protein content despite impairment of oxidative phosphorylation, thus presenting a paradox. Nevertheless, an increase in protein content was ascribed to a positive protein balance as a consequence of the impaired protein degradation and to the synthesis of chaperones protecting proteins from further denaturation [58]. More recently, the possible involvement of endoplasmic reticulum stress has been suggested, based on the ultrastructural changes found in rough endoplasmic reticulum and mitochondria [59]. Biomolecular analysis reveals that protein overexpression in cloudy swelled sheep hepatocytes was compatible with endoplasmic reticulum stress and, consequently, such a pathological state should be referred to as an early cellular defense mechanism [60], possibly acting initially as an adaptive strategy, rather than a mere degenerative change [61]. The cloudy swelling appearance is related to dilated/swollen mitochondria and, interestingly, in an earlier study, PFOA increased mitochondrial membrane permeability, leading to impaired aerobic ATP production and liver glycogen depletion to sustain the compensatory anaerobic ATP production [62]. Furthermore, the occurrence of endoplasmic reticulum stress and of the unfolded protein response have both been described in murine hepatic cells exposed to PFOA [59]. In a mechanistic toxicity study of PFOA in zebrafish, Hagenaars and colleagues proposed mitochondrial dysfunction as an extreme, irreversible change, leading to apoptosis, also emphasizing the enhanced cell turnover both at the subcellular and cellular level [62]. Signs of enhanced turnover, both at the subcellular and cellular level, related to PFOA exposure class were documented during the present survey, respectively as autophagosomes (PFOA is known to cause ER stress with the activation of authophagy [63]) and myelin figure formation and also as PCNA-positive nuclei, an indirect index of cellular mitosis [53,64]. Glycogen depletion, matrix vacuolization of mitochondria, and dilated cisternae of endoplasmic reticulum were also observed during the present survey and are strongly indicative of the possible involvement of endoplasmic reticulum stress, where the observed flocculent content inside endoplasmic reticulum may account for protein traffic impairment due to misfolded/unfolded protein retention. Endoplasmic reticulum stress may result in apoptosis in case of irreversible changes or in a conservative cell response mediated by the unfolded protein response that promote the proper protein folding in order to restore cell protein traffic [65,66,67]. This twofold, dose-dependent response is known as hormesis and is widely reported, though often neglected and misunderstood in pharmacology and toxicology [68,69].

Object complexity can be effectively approached by means of fractal analysis in terms of change in detail with respect to change in scale. As a result, fractal dimension measures the complexity, the “roughness”, whereas lacunarity measures the rotational invariance, the heterogeneity (texture) of an image [70,71,72,73,74,75]. Mathematically speaking, fractal objects have a definite fractal dimension which does not vary according to scale (so named self-similarity and scale-invariance) [72]. Though applicable to mathematical objects, such definition does not apply to natural objects where self-similarity properties are present on average and only in a limited scale range, referring more properly to “statistical self-similarity”, “partial self-similarity” or “self-affinity” [70,71,76]. Interestingly, fractal box counting applies also to real objects not strictly self-similar [77], not being the latter property mandatory for fractal analysis according to recognized authors [70,76,78]. Fractal analysis has proven useful to deal with complexity in bio-medical imaging and diagnosis [73,74,75] and has also been applied to fish histology [18,79,80,81]. During the present survey, grayscale differential box counting was utilized to assess the complexity variation of the microscopic texture in liver of carp due to PFOA exposure. Grayscale differential box counting is performed by projecting two-dimensional grayscale images into a pseudo-three dimensional space, where the third dimension is represented by the intensity (namely the gray value) of each pixel. The differential box counting method relies on the difference in pixel intensity within a box [54]. Such a method was adopted because it is better suited for texture analysis, referring fractal dimension and lacunarity, respectively, to complexity and rotational invariance of the grayscale distributional pattern (namely the texture), compared to fractal analysis applied to segmented images, where pixels are reduced to the binary logic (1–0; on–off; existence–non-existence) [33,45,48]. In a previous study on the same experimental material, texture analysis was assessed by means of other computation methods, and the extracted and selected features were analyzed using Linear Discriminant Analysis to assess their discriminative power with respect to the exposure classes (untreated, low dosage, high dosage) [15]. It was also noted that the increase in the PFOA liver concentration was associated with an increase in fractal dimension and lacunarity (grayscale complexity), preliminarily measured by means of grayscale differential box counting, and conversely with a decrease in Sum Entropy (the disorder of a vector form the gray level co-occurrence matrix), though the possible biological significance was not specifically addressed [33]. Basing upon the theory of information, and referring to a system, the complexity is the amount of information necessary to describe the system [82]. According to the theory of communication, the more the entropy of a system increases, the more the related information decreases [82,83], and therefore the relationship of the previously reported negative correlation (namely fractal dimension and lacunarity increase and Sum Entropy decrease) appears meaningful.

Recently, the traditional tendency to dichotomize health and disease, physiological and pathological, has been revisited, leading to the idea that disease should be considered as a loss of inherent complexity, rather than a loss of order. Accordingly, fractal analysis has been used to assess how complexity was reduced as a consequence of pathological changes in biological systems assumed to behave as “chaotic” systems [84,85]. In the present survey, a significant complexity increase was associated with the modification of liver microscopic texture due to PFOA exposure. Nevertheless, this apparently contradictory behavior should be related to reversible changes (cloudy swelling), possibly representing a primarily adaptive strategy rather than a slight, reversible form of degeneration as traditionally proposed, to cope with PFOA challenge as suggested by other authors [61]. As noted earlier, protein overexpression has been reported in sheep liver during cloudy swelling [60] and this fact is compatible with an informative increase, and accordingly with a decrease in entropy and an increase in complexity as reported during the present survey. Moreover, the possible occurrence of a compensatory, adaptive unfolded protein response and hence of a hormesis [86] should be considered, where a true degenerative, irreversible response leading to apoptosis overcomes the previous, leading to a substantial and true pathological loss of complexity. 

## 5. Conclusions

The application of grayscale differential box counting showed a clear complexity increase associated, both structurally and ultrastructurally, to “cloudy swelling” as a consequence of PFOA exposure. This complexity increase agreed with previous outcomes on the same experimental material, obtained by means of another texture analysis method, where a decrease in disorder in the grayscale pattern was similarly associated with PFOA exposure [15]. Though cloudy swelling has been considered as a slight, reversible form of cell degeneration, it should be regarded as an initial adaptive strategy to cope with PFOA challenge, possibly related to endoplasmic reticulum stress and unfolded protein response, thereby linking the morphological (grayscale pattern) with the functional (protein overexpression) complexity increase.

## Figures and Tables

**Table 1:** Fractal dimension and lacunarity according to exposure group.

		N	Mean	Std. Dev.	Contrast 1 Ctr vs. Low PFOA		Contrast 2 Ctr vs. High PFOA		Contrast 3 Low PFOA vs. High PFOA	
**Fractal dimension**	**Ctr**	10	2.172	0.016	1	*p* > 0.05	1	*p* < 0.01	0	*p* < 0.01
	**Low PFOA**	10	2.172	0.010	−1		0		1	
	**High PFOA**	10	2.199	0.014	0		−1		−1	
**Lacunarity**	**Ctr**	10	0.011	0.002	1	*p* > 0.05	1	*p* < 0.01	0	*p* < 0.01
	**Low PFOA**	10	0.011	0.002	−1		0		1	
	**High PFOA**	10	0.014	0.002	0		−1		−1	

**Table 2:** Liver PFOA concentration, liver mass and number of proliferating cell nuclear antigen (PCNA)-immunoreactive nuclei according to exposure group [15].

		N	Mean	Std. Dev.	Contrast 1 Ctrl vs. Low PFOA		Contrast 2 Ctrl vs. High PFOA		Contrast 3 Low PFOA vs. High PFOA	
**Liver PFOA concentration (ng·g^−1^)**	**Ctr**	10	0.2 *	0.0	1	-	1	*p* < 0.01	0	*p* < 0.01
	**Low PFOA**	10	0.2 *	0.0	−1		0		1	
	**High PFOA**	10	28.4	5.5	0		−1		−1	
**Liver mass (g)**	**Ctr**	10	2.7	0.7	1	*p* < 0.05	1	*p* > 0.05	0	*p* < 0.05
	**Low PFOA**	10	3.6	1.0	−1		0		1	
	**High PFOA**	10	2.6	0.8	0		−1		−1	
**PCNA-immunoreactive nuclei**	**Ctr**	10	17.2	10.4	1	*p* = 0.059	1	*p* = 0.057	0	*p* > 0.05
	**Low PFOA**	10	47.2	43.3	−1		0		1	
	**High PFOA**	10	45.9	40.9	0		−1		−1	

* Values under the limit of detection (LOD) were set to LOD·2^−1^.
